# Clinical risk factors associated with coronary slow flow: a meta-analysis

**DOI:** 10.1186/s12872-026-06373-9

**Published:** 2026-07-29

**Authors:** Guangrong Zi, Guofu Zhu

**Affiliations:** Cardiology Department, the Second Affiliated Hospital of Kunming Medical University, Kunming, Yunnan Province 650000 China

**Keywords:** Coronary slow flow phenomenon, Risk factors, Case–control studies, Meta-analysis

## Abstract

**Objective:**

To investigate factors associated with coronary slow flow (CSF) using a meta-analysis and to provide evidence-based support for the prevention and management of coronary artery disease.

**Methods:**

Relevant studies published between January 1, 2015, and December 31, 2025, were retrieved from CNKI, CBM, Wanfang, VIP, PubMed, and Embase using the search terms “coronary slow flow,” “coronary slow flow phenomenon,” “coronary slow flow syndrome,” “related factors,” “influencing factors,” and “risk factors.” Case–control studies that met the predefined inclusion and exclusion criteria were included. Study quality was assessed using the Newcastle–Ottawa Scale (NOS), with studies scoring ≥ 7 considered high quality. Meta-analysis was performed using RevMan version 5.3. This study was registered with PROSPERO (ID: CRD420261334323).

**Results:**

A total of 41 studies were included in the systematic review. Quantitative meta-analysis was performed only for risk factors reported in a sufficient number of studies with comparable definitions and extractable data. The meta-analysis suggested that smoking (OR = 1.21, 95% CI: 1.13–1.29), higher body mass index (OR = 1.20, 95% CI: 1.06–1.36), male sex (OR = 1.51, 95% CI: 1.26–1.81), hypertension (OR = 2.32, 95% CI: 1.27–4.26), elevated homocysteine (OR = 1.55, 95% CI: 1.13–2.13), elevated high-sensitivity C-reactive protein (OR = 1.62, 95% CI: 1.36–1.94), and higher uric acid levels (OR = 1.90, 95% CI: 1.47–2.46) were associated with higher odds of CSF. In contrast, higher high-density lipoprotein cholesterol (HDL-C) levels were associated with lower odds of CSF (OR = 0.45, 95% CI: 0.38–0.55).

**Conclusion:**

Coronary slow flow (CSF) may be associated with multiple clinical and biochemical factors. Smoking, increased body mass index, male sex, hypertension, and elevated levels of homocysteine, high-sensitivity C-reactive protein, and uric acid were associated with higher odds of CSF, whereas higher HDL-C levels were associated with lower odds of CSF. These findings suggest that unhealthy lifestyle behaviors, metabolic abnormalities, and inflammatory responses may contribute to the development of CSF. Although many CSF-associated factors are potentially modifiable, early interventions—particularly smoking cessation, blood pressure and weight control, and improvement of metabolic and inflammatory status—may help reduce the likelihood of CSF. However, these associations should be interpreted cautiously because moderate-to-high heterogeneity remained in several pooled analyses. Future high-quality prospective studies are needed to clarify the relative importance of these factors, examine potential causal relationships, and evaluate targeted interventions.

**Supplementary Information:**

The online version contains supplementary material available at https://doi.org/10.1186/s12872-026-06373-9.

## Introduction

Coronary slow flow (CSF) is a distinct angiographic phenomenon characterized by delayed opacification and clearance of contrast medium in the distal coronary arteries, despite the absence of significant epicardial coronary stenosis or the presence of only mild luminal narrowing. This condition is also known as “cardiac syndrome Y” [1]. The diagnosis of CSF primarily relies on quantitative methods, such as the corrected thrombolysis in myocardial infarction frame count (cTFC). CSF is considered a cardiovascular syndrome primarily characterized by coronary microvascular dysfunction and/or endothelial dysfunction [2]. Epidemiological studies indicate that the prevalence of CSF among patients undergoing diagnostic coronary angiography ranges from approximately 1% to 7%. This prevalence may increase to approximately 10% among patients without significant coronary stenosis on angiography. A retrospective cohort study of 23,159 patients undergoing coronary angiography reported a CSF incidence of 2.65%, suggesting that CSF is not an uncommon clinical finding [3]. In recent years, the detection of CSF has increased with the widespread use of cardiovascular interventional techniques and growing recognition of coronary microvascular disorders. This trend suggests that CSF may represent a distinct clinical entity with specific clinical and pathophysiological characteristics. Although CSF is not associated with typical obstructive coronary artery disease, it has been linked to various adverse prognostic events. Patients with CSF frequently present with recurrent chest pain and chest tightness, with recurrence rates ranging from 20% to 80%; these rates are substantially higher than those observed in individuals with normal coronary flow. This condition may increase the risk of emergency department visits and rehospitalization. Several follow-up studies have suggested that patients with CSF may have a higher risk of major adverse cardiovascular and cerebrovascular events (MACCE) than individuals with normal coronary flow. After adjustment for traditional cardiovascular risk factors, CSF has been reported as an independent predictor of major adverse cardiovascular events (MACE), with hazard ratios ranging from approximately 1.5 to 5.9 [4]. These adverse outcomes include cardiac death, myocardial infarction, malignant arrhythmias, stroke, and heart failure. Some patients may require repeated coronary angiography or even revascularization procedures, leading to substantial healthcare and socioeconomic burdens. Notably, some prospective studies with relatively small sample sizes have suggested that, after excluding patients with prior myocardial infarction or structural myocardial injury, certain individuals with CSF did not experience increased mortality or acute coronary syndrome during a 5-year follow-up period. In these patients, left ventricular systolic function and myocardial strain parameters remained relatively stable. These findings suggest that CSF may not universally increase mortality but may be more commonly characterized by recurrent chest pain, reduced quality of life, and an elevated risk of MACE [5]. Collectively, these findings highlight the complexity of the natural history and long-term prognosis of CSF and suggest that its clinical significance cannot be fully understood solely from the perspective of traditional obstructive coronary artery disease. Several mechanisms have been proposed to contribute to the development and progression of CSF. These mechanisms include structural remodeling and spasm of the coronary microcirculation, diffuse early atherosclerosis, endothelial dysfunction, inflammatory activation, oxidative stress, increased platelet reactivity, hypercoagulability, and autonomic nervous system dysregulation [6]. Clinical observations further suggest that patients with CSF frequently exhibit traditional cardiovascular risk factors, such as smoking, obesity, hypertension, and dyslipidemia, as well as features of metabolic syndrome and elevated inflammatory markers. However, findings across studies remain inconsistent, and high-quality evidence from systematic reviews and meta-analyses quantifying these associations remains limited. Therefore, we synthesized evidence from case–control studies conducted over the past decade to quantitatively evaluate the associations between CSF and traditional cardiovascular risk factors, metabolic parameters, and inflammatory biomarkers. This study aims to provide evidence-based support for risk stratification and comprehensive prevention strategies in patients with CSF, while also contributing to a better understanding of its underlying pathophysiological mechanisms and clinical heterogeneity.

## Data and methods

### Literature search

A comprehensive literature search was conducted in the following electronic databases: China National Knowledge Infrastructure (CNKI), China Biological Medicine Database (CBM), Wanfang Data Knowledge Service Platform, VIP Chinese Science and Technology Journals Database, PubMed, and Embase. All databases were searched from January 1, 2015, to December 31, 2025. The search strategy incorporated both controlled vocabulary terms (e.g., MeSH terms in PubMed and Emtree terms in Embase) and free-text keywords. The following search terms were used: “coronary slow flow” OR “coronary slow flow phenomenon” OR “coronary slow flow syndrome,” combined with “risk factors” OR “related factors” OR “influencing factors.” The search strategy was adapted for each database as appropriate. In addition, the reference lists of relevant articles were manually screened to identify any additional eligible studies.

### Literature inclusion and exclusion criteria

All risk factors included in the quantitative meta-analysis were treated as continuous variables in the original studies. Adjusted odds ratios (ORs) and corresponding 95% confidence intervals were extracted directly from the multivariable regression models reported in the original articles. None of the included studies converted these risk factors into categorical variables for risk estimation. Therefore, the pooled estimates were based on adjusted ORs for continuously measured exposures. Although this approach improved comparability across studies, residual heterogeneity due to differences in covariate adjustment could not be completely excluded. To minimize potential patient overlap, the study setting, hospital or institution, recruitment period, author group, sample size, and baseline characteristics of all included studies were further reviewed, particularly for studies from the same region or institution. When studies appeared to originate from overlapping cohorts, the study with the larger sample size, more complete data, or clearer reporting was preferentially included. Studies with suspected duplicate populations were excluded or retained only after the study periods, institutions, or patient sources were confirmed to be sufficiently distinct.

#### Inclusion criteria

- ① Study design: Case–control studies were included. This meta-analysis was restricted to case–control studies to improve methodological comparability across included studies. The currently available evidence on factors associated with coronary slow flow (CSF) is predominantly derived from case–control investigations, whereas prospective cohort studies remain limited. Given the observational nature of the available literature, case–control studies were considered appropriate for evaluating associations between CSF and potential clinical or biochemical factors. Limiting the analysis to this study design allowed a more focused and consistent synthesis of the most relevant evidence currently available.
- ② Study population: The case group consisted of patients diagnosed with coronary slow flow (CSF) by coronary angiography (CAG) in hospital settings. According to the criteria proposed by Gibson et al., CSF was defined as a corrected TIMI frame count (CTFC) of ≥ 27 frames in at least one coronary artery at a recording speed of 30 frames per second. The control group comprised patients from the same institutions who underwent CAG during the same period and had a CTFC of < 27 frames, indicating normal coronary flow.
- ③ Publication period and exposure factors: Studies published between January 1, 2015, and December 31, 2025, from both Chinese and international sources were included. Eligible studies reported associations between CSF and at least one of the following factors: smoking, body mass index (BMI), male sex, homocysteine, high-sensitivity C-reactive protein (hs-CRP), high-density lipoprotein cholesterol (HDL-C), uric acid, hypertension, serum lipoprotein-associated phospholipase A2 (Lp-PLA2), endothelin-1 (ET-1), triglyceride–glucose index (TyG), neutrophil-to-monocyte ratio, plasma thrombomodulin, systemic immune-inflammation index (SII), anxiety, and depression.
- ④ Outcome reporting: Eligible studies provided sufficient data to directly extract or calculate odds ratios (ORs), corresponding 95% confidence intervals (CIs), and standard errors (SEs).

#### Exclusion criteria

- ① Duplicate publications were excluded.
- ② Studies with unclear outcome measures or inappropriate statistical methods were excluded.
- ③ Studies without a control group, with incomplete baseline data, or with excessive loss to follow-up were excluded.
- ④ Review articles, systematic reviews, meta-analyses, animal studies, and other non-original research were excluded.
- ⑤ Studies in which the definitions of risk factors differed substantially from generally accepted criteria or from those used in the majority of included studies were excluded.
- ⑥ Studies in which the sample size of either the case group or the control group was fewer than 50 participants were excluded.

### Literature screening process

**Fig. 1:**
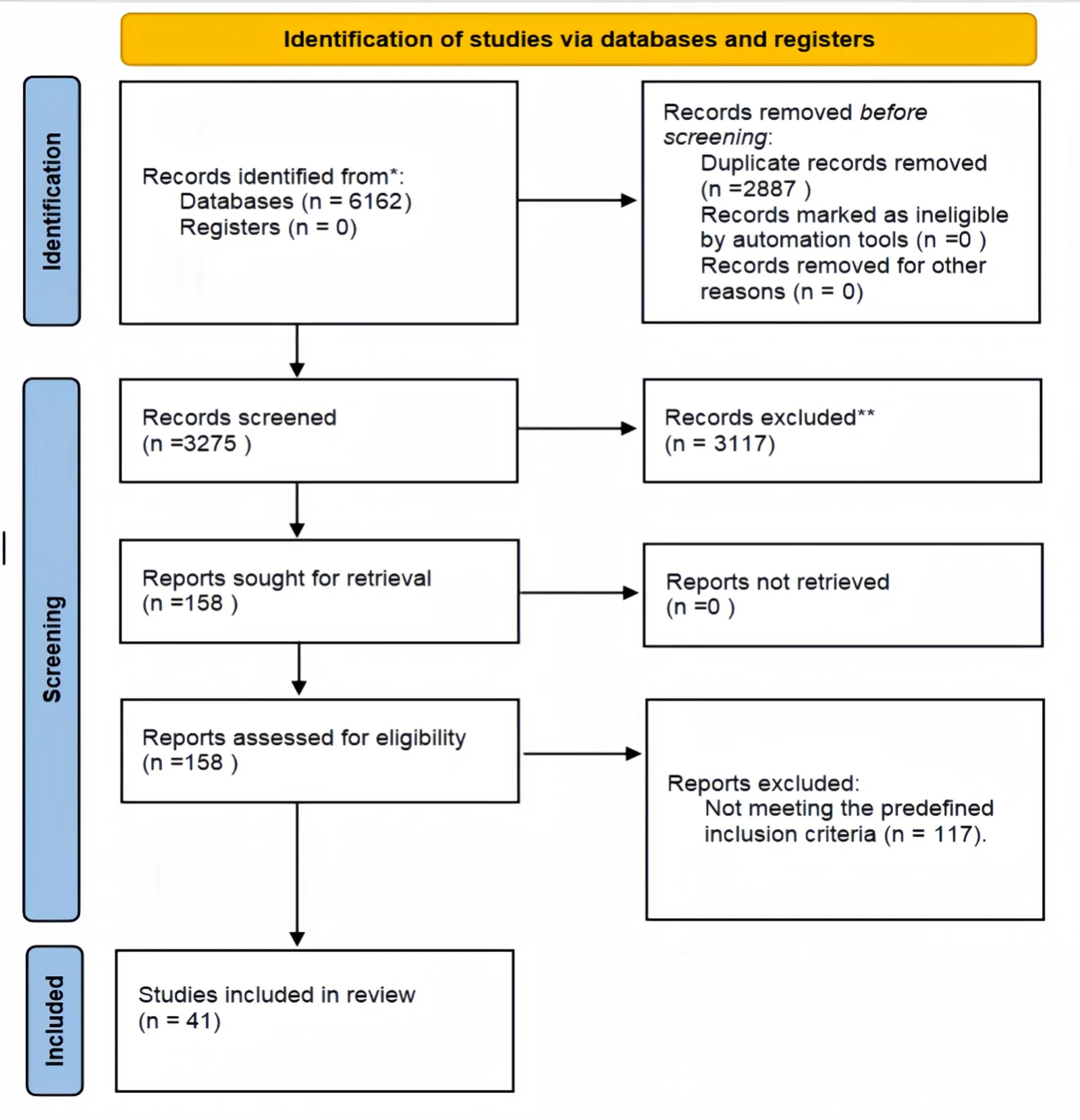
PRISMA 2020 flow diagram of the study selection process

A total of 6,162 records were identified through database searches. After 2,887 duplicates were removed, 3,275 records remained for title and abstract screening. Of these, 3,117 records were excluded, and 158 reports were retrieved for full-text assessment. After full-text evaluation based on the predefined inclusion and exclusion criteria, 117 reports were excluded. Ultimately, 41 studies were included in the systematic review [7–48] (see Fig. 1).

### Essential characteristics and quality assessment of included literature

Among the 41 included studies, 33 were published in Chinese and 8 in English; all employed a case–control design. The studies were published between 2015 and 2025 and included 4,625 participants in the CSF group and 5,334 participants in the control group. The methodological quality of the included observational studies was assessed using the Newcastle–Ottawa Scale (NOS). The NOS primarily evaluates three domains: selection bias, comparability between study groups, and the adequacy of outcome assessment [49]. Higher total scores indicate better methodological quality. Quality assessment was performed independently by two reviewers, and any discrepancies were resolved through discussion or consultation with a third reviewer. All included studies achieved a NOS score of ≥ 7 stars, indicating generally high methodological quality (see Table 1).

**Table 1:** Characteristics and quality assessment of the included studies

First Author	Year	Study type	Number of cases	Number of controls	Control the population source	Total Score of the NOS	Risk factor
Li Bing	2019	case-control study	69	60	The Third Affiliated Hospital of Inner Mongolia Medical University (China)	8 Stars	1, 2, 4, 8, 9
Wang Shuai	2016	case-control study	560	325	Xijing Hospital, Air Force Medical University (China)	8 Stars	3, 5, 8, 10, 11,34
Su Yang	2021	case-control study	83	108	The Affiliated Hospital of Qingdao University (China)	7 Stars	3, 12
Lei Jing	2022	case-control study	118	112	Chenzhou No. 1 People’s Hospital (China)	8 Stars	13, 14
Zhang Yuxin	2015	case-control study	84	77	The First Affiliated Hospital of Zhengzhou University (China)	8 Stars	31
Chen Yun	2024	case-control study	104	126	The First Affiliated Hospital of Henan University of Chinese Medicine (China)	7 Stars	1, 16, 17, 18
Yu Jiang	2024	case-control study	151	152	People’s Liberation Army General Hospital (China)	8 Stars	1, 19, 20, 21, 22,23
Zhao Ziwei	2020	case-control study	429	432	Beijing Anzhen Hospital, Capital Medical University (China)	7 Stars	1, 3, 24
Zhu Yanan	2023	case-control study	188	106	Langfang City Traditional Chinese Medicine Hospital (China)	8 Stars	3, 6, 25, 26
Wang Jing	2015	case-control study	56	55	The Second Affiliated Hospital of Xuzhou Medical University (China)	8 Stars	27
Zhang Zhiqiang	2025	case-control study	57	57	Langfang Hospital of Traditional Chinese Medicine (China)	8 Stars	2, 28, 29, 30
Yang Qianqian	2017	case-control study	139	232	Affiliated Hospital of Binzhou Medical University (China)	7 Stars	19, 32, 33, 34, 35
Lu Cong	2018	case-control study	183	124	Chengdu First People’s Hospital (China)	7 Stars	3, 46
Yao Kunmei	2023	case-control study	66	66	Guilin Medical University (China)	7 Stars	6, 36
Xu Min	2016	case-control study	89	87	Beijing Luhe Hospital, Capital Medical University (China)	8 Stars	1, 5, 37
Yang Fan	2016	case-control study	80	100	The First Affiliated Hospital of Zhengzhou University (China)	8 Stars	4, 38
Chen Bo	2021	case-control study	65	183	Putuo District People’s Hospital of Zhoushan (China)	8 Stars	2, 8, 39
Wang Ting	2019	case-control study	100	100	People’s Hospital of Xinjiang Uygur Autonomous Region (China)	8 Stars	2, 40, 41
Run Jie	2018	case-control study	54	54	Affiliated Hospital of North China University of Science and Technology (China)	8 Stars	42, 43
Qin Gang	2017	case-control study	60	60	First Hospital of Shanxi Medical University (China)	7 Stars	31, 44
Wang Li	2021	case-control study	87	228	Beijing Huaxin Hospital, Tsinghua University (China)	8 Stars	1, 37, 44
Wang Jirong	2015	case-control study	50	50	Shanxi Provincial People’s Hospital (China)	8 Stars	4, 9
Kong Qianwen	2020	case-control study	80	80	Zhoushan Hospital (China)	8 Stars	5, 8, 38, 46, 47
Ge Hailong	2015	case-control study	83	96	Beijing Anzhen Hospital, Capital Medical University (China)	8 Stars	1, 4, 48
Wang Lei	2019	case-control study	53	56	Beijing Anzhen Hospital, Capital Medical University (China)	8 Stars	6, 49
Ren Yingang	2016	case-control study	82	184	Tangdu Hospital, Air Force Medical University (China)	7 Stars	42
Yang Pengkang	2020	case-control study	62	60	Xi’an First Hospital (China)	7 Stars	50, 51
Tuo Buxiong	2018	case-control study	58	126	The 986th Hospital of the People’s Liberation Army (China)	8 Stars	52
Han Wei	2015	case-control study	82	184	Tangdu Hospital, Air Force Medical University (China)	8 Stars	53
Shi Zhifang	2023	case-control study	98	108	Shijiazhuang Third Hospital (China)	8 Stars	7, 54, 55
Li Shijing	2018	case-control study	146	101	Mentougou District Hospital, Beijing (China)	8 Stars	6, 7, 34, 56, 57
Tu Guang	2023	case-control study	80	80	Suizhou Hospital, Hubei University of Medicine (China)	7 Stars	2, 3, 58
Wei Xing	2023	case-control study	212	131	Hefei Hospital, Anhui Medical University (China)	8 Stars	4, 59
Ramy Raymond Elias	2025	case-control study	60	60	Ain Shams University(Egypt)	8 Stars	1, 60, 61, 62, 63
Kenan Toprak	2024	case-control study	130	130	Faculty of Medicine, Harran University(Turkey)	8 Stars	1, 2, 20, 64, 65
Mehmet Serkan Cetin	2015	case-control study	200	200	Turkey Yuksek Ihtisas Education & Research Hospital(Turkey)	8 Stars	1, 7, 66, 67
Ahmed A. Elamragy	2019	case-control study	50	50	University of Bologna(Italy)	8 Stars	3, 41, 60
Nan Jing	2025	case-control study	69	586	Beijing Tiantan Hospital, Capital Medical University (China)	8 Stars	18
Zhang Yunbo	2025	case-control study	73	73	The Second Affiliated Hospital of Kunming Medical University (China)	8 Stars	5, 68, 69
Hamidreza Sanati	2016	case-control study	75	75	Iran University of Medical Sciences (Iran)	8 Stars	2, 5, 7, 70
Ahmed Shawky Shereef	2024	case-control study	60	60	Faculty of Medicine, Zagazig University(Egypt)	8 Stars	1, 45,68

1. Smoking,2. Body mass index (BMI),3. Male sex,4. Homocysteine,5. High-density lipoprotein cholesterol (HDL-C),6. High-sensitivity C-reactive protein (hs-CRP),7. Hypertension,8. Uric acid,9. Endothelin-1 (ET-1),10. Smoking duration,11. Heart rate,12. High-sensitivity C-reactive protein-to-albumin ratio (hs-CRP/Alb),13. Tp–Te interval,14. Tp–Te/QT ratio,15. Hematocrit,16. Low-density lipoprotein cholesterol (LDL-C),17. Systemic immune-inflammation index (SII),18. Triglyceride–glucose index (TyG index),19. Diabetes mellitus,20. Hyperlipidemia,21. Coronary sinus diameter,22. Left atrial left–right diameter,23. Peak mitral inflow velocity (E wave),24. Red blood cell count,25. Lipoprotein(a) [Lp(a)],26. Cystatin C,27. Mean corpuscular volume (MCV),28. Mitral inflow E/A ratio < 1,29. P-selectin,30. B-type natriuretic peptide (BNP),31. Neutrophil-to-lymphocyte ratio (NLR),32. Lymphocyte-to-monocyte ratio (LMR),33. Neutrophil-to-monocyte ratio (NMR),34. Age,35. Total bile acids,36. Growth differentiation factor-15 (GDF-15),37. Red cell distribution width (RDW),38. Fragmented QRS (fQRS),39. Hypertension combined with diabetes mellitus,40. Anxiety,41. Depression,42. Soluble lectin-like oxidized low-density lipoprotein receptor-1 (sLOX-1),43. Leptin,44. Eosinophil count,45. LV global longitudinal strain,46. Hematocrit,47. Platelet distribution width (PDW),48. D-dimer,49. MicroRNA-126 (miR-126),50. MicroRNA-155 (miR-155),51. Erythrocyte aggregation index,52. Plasma thrombomodulin,53. Serum endocan,54. Serum platelet-activating factor (PAF),55. Serum lipoprotein-associated phospholipase A2 (Lp-PLA2),56. Female sex,57. Fundus arteriosclerosis grade > 2,58. Neutrophil percentage-to-albumin ratio,59. Neutrophil-to-high-density lipoprotein cholesterol ratio (NHR),60. Triglycerides,61. Serum glucose,62. Blood urea nitrogen (BUN),63. Serum osmolality,64. Atherogenic index,65. Castelli risk index II,66. Whole blood viscosity,67. White blood cell count (WBC),68. Global longitudinal strain (GLS),69. Global left ventricular perfusion index,70. Hemoglobin

### Data processing and analysis

The meta-analysis was conducted using Review Manager (RevMan) version 5.3 software. Between-study heterogeneity was assessed using Cochran’s Q test and quantified using the I² statistic [50]. When *P* > 0.05 and I² < 50%, heterogeneity was considered not statistically significant, and a fixed-effect model was applied. When *P* ≤ 0.05 and I² ≥ 50%, substantial heterogeneity was assumed, and a random-effects model was used. Sensitivity analyses were performed using a leave-one-out approach to evaluate the robustness of the pooled results and to identify potential sources of heterogeneity. Specifically, each study was sequentially excluded, and the meta-analysis was repeated to assess the impact of individual studies on the overall effect size and heterogeneity estimates. If substantial changes in heterogeneity or effect estimates were observed after exclusion of a particular study, that study was considered a potential source of heterogeneity. The choice of the final effect model (fixed-effect or random-effects) was further determined based on the results of the sensitivity analysis.

Sensitivity analyses were performed by sequentially excluding one study at a time or removing studies with high heterogeneity to assess the robustness of the pooled results. The primary conclusions were based on the full dataset, and sensitivity analyses were used only to evaluate stability rather than to redefine the main effect estimates. Publication bias was initially assessed by visual inspection of funnel plots. Statistical tests for publication bias, including Egger’s test and Begg’s test, were considered only when at least 10 studies were available for a given outcome. In this study, only the original pooled analysis of smoking included 11 studies and was therefore eligible for statistical publication bias testing. However, after sensitivity analysis, the smoking analysis included only eight studies; therefore, the funnel plot was interpreted as exploratory, and Egger’s and Begg’s tests were not repeated. For the remaining outcomes, publication bias tests were not performed because fewer than 10 studies were available, resulting in limited statistical power.

## Results

### Meta-analysis of associated factors

**Fig. 2:**
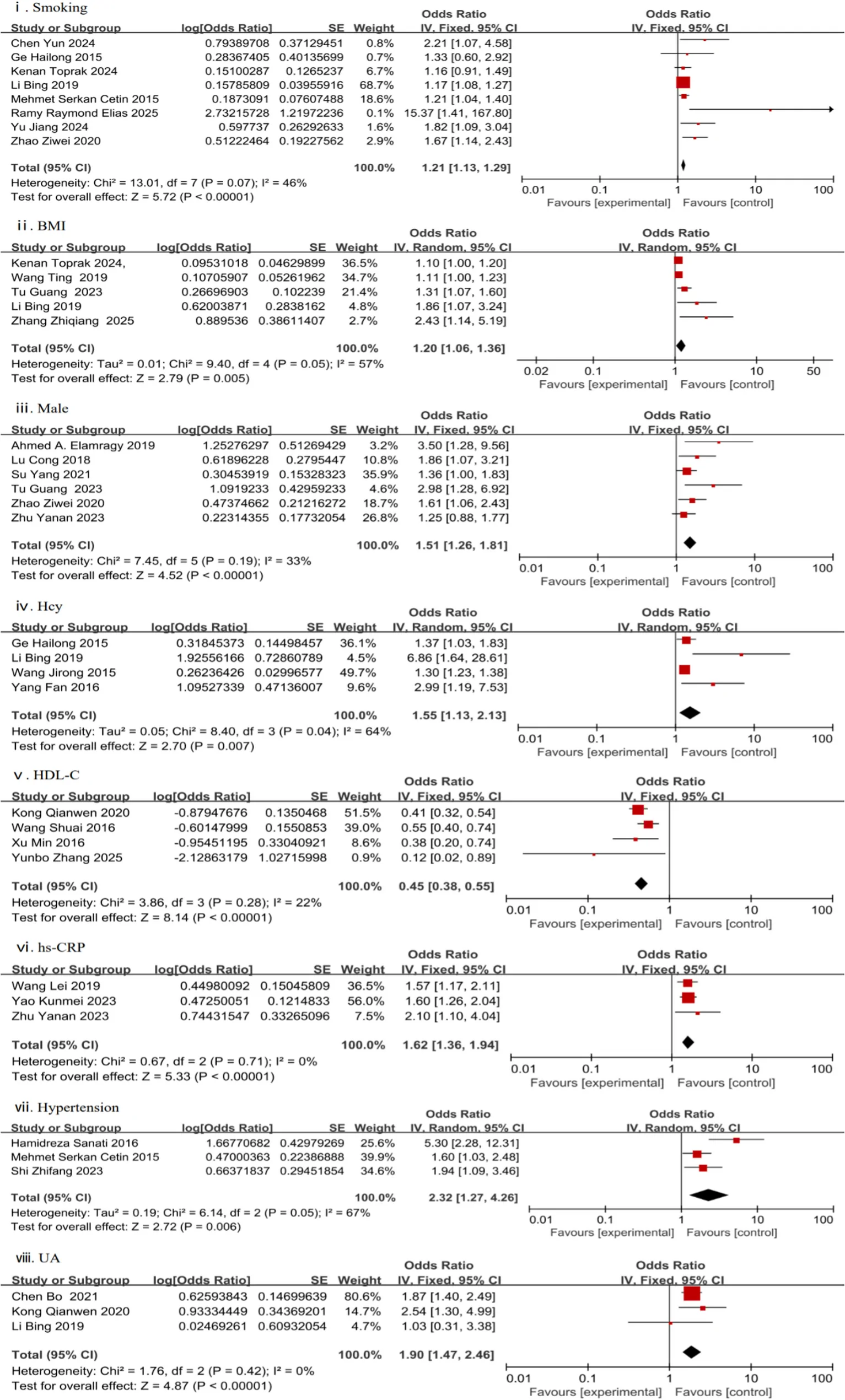
Forest plot of the meta-analysis of factors associated with coronary slow flow

A meta-analysis was conducted to evaluate the associations between coronary slow flow (CSF) and selected clinical and biochemical factors, including smoking, body mass index (BMI), male sex, homocysteine, high-density lipoprotein cholesterol (HDL-C), high-sensitivity C-reactive protein (hs-CRP), hypertension, and uric acid. Based on the overall pooled analyses, higher HDL-C levels were associated with lower odds of CSF, whereas smoking, increased BMI, elevated homocysteine, elevated hs-CRP, hypertension, and higher uric acid levels were associated with higher odds of CSF. The association between male sex and CSF should be interpreted cautiously because its statistical significance was affected by sensitivity analyses, suggesting potential instability and between-study heterogeneity (Table 2) (Fig. 2).

**Table 2:** Meta-analysis of factors associated with coronary slow flow

Risk factor	Analysis Phase	Number of studies	Odds Ratio	95%CI	Heterogeneity test			Overall effect test	
					Q	*P*	I²(%)	Z-value	*P* -value
Ⅰ. Smoking	Before Sensitivity Analysis	11	1.67	1.34 ~ 2. 07	42.37	< 0.01	76	4.59	< 0.05
	After Excluding [Xu Min 2016; Wang Li 2021; Ahmed Shawky Shereef 2024]	8	1.21	1.13 ~ 1.29	13.01	0.07	46	5.72	< 0.05
Ⅱ. BMI	Before Sensitivity Analysis	7	1.30	1.01 ~ 1.67	116.31	< 0.01	95	2.05	< 0.05
	After Excluding [Hamidreza Sanati. 2016; Chen Bo 2021]	5	1.20	1.06 ~ 1.36	9.40	0.05	57	2.79	< 0.05
Ⅲ. Male	Before Sensitivity Analysis	7	1.15	0.99 ~ 1.35	42.39	< 0.01	86	1.80	0.07
	After Excluding [Wang Shuail 2016]	6	1.51	1.26 ~ 1.81	7.45	0.19	33	4.52	< 0.05
Ⅳ. Hcy	Before Sensitivity Analysis	5	1.29	1.09 ~ 1.54	25.67	< 0.01	84	2.93	< 0.05
	After Excluding [Wei Xing 2023]	4	1.55	1.13 ~ 2.13	8.40	0.04	64	2.70	< 0.05
Ⅴ. HDL-C	Before Sensitivity Analysis	5	0.76	0.69 ~ 0.83	40.82	< 0.01	90	5.72	< 0.05
	After Excluding [Hamidreza Sanati. 2016]	4	0.45	0.38 ~ 0.55	3.86	0.28	22	8.14	< 0.05
Ⅵ.hs-CRP	Before Sensitivity Analysis	4	1.46	1.23 ~ 1.73	16.24	< 0.01	82	4.36	< 0.05
	After Excluding [Li Shijing 2018]	3	1.62	1.36 ~ 1.94	0.67	0.71	0	5.33	< 0.05
Ⅶ. Hypertension	Before Sensitivity Analysis	4	1.66	0.76 ~ 3.64	21.62	< 0.01	86	1.26	0.21
	After Excluding [Li Shijing 2018]	3	2.32	1.27 ~ 4.26	6.14	0.05	67	2.72	< 0.05
Ⅷ.UA	Before Sensitivity Analysis	4	1.50	0.90 ~ 2.49	25.43	< 0.01	88	1.54	< 0.12
	After Excluding [Wang Shuai 2016]	3	1.90	1.47 ~ 2.46	1.76	0.42	0	4.87	< 0.05

I. A meta-analysis was conducted to evaluate smoking as a factor associated with coronary slow flow (CSF). The initial heterogeneity assessment indicated substantial between-study heterogeneity (I² = 76%, *P* < 0.05). Sensitivity analysis suggested the studies by Xu et al. (2016), Wang et al. (2021), and Shereef et al. (2024) as potential sources of heterogeneity. After excluding these studies, the meta-analysis was repeated, and heterogeneity was reduced (I² = 46%, *P* = 0.07), suggesting moderate heterogeneity. Given that the heterogeneity was below the conventional threshold (I² < 50%), a fixed-effect model was applied for the sensitivity analysis. The pooled results suggested that smoking may be associated with increased odds of CSF (Z = 5.72, *P* < 0.05). Specifically, smoking was associated with 1.21-fold higher odds of CSF (95% CI: 1.13–1.29).
II. A meta-analysis was conducted to evaluate body mass index (BMI) as a factor associated with coronary slow flow (CSF). The initial heterogeneity assessment revealed substantial between-study heterogeneity (I² = 95%, *P* < 0.05). Sensitivity analysis suggested the studies by Sanati et al. (2016) and Chen et al. (2021) as potential sources of heterogeneity. After excluding these studies, the meta-analysis was repeated, and heterogeneity was reduced (I² = 57%, *P* = 0.05), suggesting moderate heterogeneity. Given that the heterogeneity was above the conventional threshold (I² > 50%), a random-effects model was applied for the sensitivity analysis. The pooled results suggested that BMI may be associated with increased odds of CSF (Z = 2.79, *P* < 0.05). Specifically, BMI was associated with 1.20-fold higher odds of CSF (95% CI: 1.06–1.36).
III. A meta-analysis was conducted to evaluate male sex as a factor associated with coronary slow flow (CSF). The initial heterogeneity assessment indicated substantial between-study heterogeneity (I² = 86%, *P* < 0.05). Sensitivity analysis suggested the study by Wang et al. (2016) as a potential source of heterogeneity. After excluding this study, the meta-analysis was repeated. The heterogeneity was markedly reduced (I² = 33%, *P* = 0.19), indicating no significant residual heterogeneity. Based on these findings, a fixed-effect model was applied for the sensitivity analysis. The pooled results suggested that male sex may be associated with increased odds of CSF (Z = 4.52, *P* < 0.05). Specifically, men had 1.51-fold higher odds of CSF than women (95% CI: 1.26–1.81).
IV. A meta-analysis was conducted to evaluate homocysteine as a factor associated with coronary slow flow (CSF). The initial heterogeneity assessment indicated substantial between-study heterogeneity (I² = 84%, *P* < 0.05). Sensitivity analysis suggested the study by Wei et al. (2023) as a potential source of heterogeneity. After excluding this study, the meta-analysis was repeated, and heterogeneity was reduced (I² = 64%, *P* = 0.04), suggesting moderate heterogeneity. Given that the heterogeneity was above the conventional threshold (I² > 50%), a random-effects model was applied for the sensitivity analysis. The pooled results suggested that homocysteine may be associated with increased odds of CSF (Z = 2.70, *P* < 0.05). Specifically, higher levels of homocysteine were associated with 1.55-fold higher odds of CSF (95% CI: 1.13–2.13).
V. A meta-analysis was conducted to evaluate high-density lipoprotein cholesterol (HDL-C) as a factor associated with coronary slow flow (CSF). The initial heterogeneity assessment indicated substantial between-study heterogeneity (I² = 90%, *P* < 0.05). Sensitivity analysis suggested the study by Sanati et al. (2016) as a potential source of heterogeneity. After excluding this study, the meta-analysis was repeated. The heterogeneity was reduced (I² = 22%, *P* = 0.28), indicating no significant residual heterogeneity. Based on these findings, a fixed-effect model was applied for the sensitivity analysis. The pooled results suggested that higher HDL-C levels may be associated with lower odds of CSF (Z = 8.14, *P* < 0.05). Specifically, elevated HDL-C levels were associated with 0.45-fold odds of CSF, suggesting a potential protective association (95% CI: 0.38–0.55).
VI. A meta-analysis was conducted to evaluate high-sensitivity C-reactive protein (hs-CRP) as a factor associated with coronary slow flow (CSF). The initial heterogeneity assessment indicated substantial between-study heterogeneity (I² = 82%, *P* < 0.05). Sensitivity analysis suggested the study by Li et al. (2018) as a potential source of heterogeneity. After excluding this study, the meta-analysis was repeated. The heterogeneity was reduced (I² = 0%, *P* = 0.71), indicating no significant residual heterogeneity. Based on these findings, a fixed-effect model was applied for the sensitivity analysis. The pooled results suggested that elevated hs-CRP levels may be associated with increased odds of CSF (Z = 5.33, *P* < 0.05). Specifically, higher hs-CRP levels were associated with 1.62-fold higher odds of CSF (95% CI: 1.36–1.94).
VII. A meta-analysis was conducted to evaluate hypertension as a factor associated with coronary slow flow (CSF). The initial heterogeneity assessment indicated substantial between-study heterogeneity (I² = 86%, *P* < 0.05). Sensitivity analysis suggested the study by Li et al. (2018) as a potential source of heterogeneity. After excluding this study, the meta-analysis was repeated, and heterogeneity was reduced (I² = 67%, *P* = 0.05), suggesting moderate heterogeneity. Given that the heterogeneity was above the conventional threshold (I² > 50%), a random-effects model was applied for the sensitivity analysis. The pooled results suggested that hypertension may be associated with increased odds of CSF (Z = 2.72, *P* < 0.05). Specifically, hypertension was associated with 2.32-fold higher odds of CSF (95% CI: 1.27–4.26).
VIII. A meta-analysis was conducted to evaluate uric acid as a factor associated with coronary slow flow (CSF). The initial heterogeneity assessment indicated substantial between-study heterogeneity (I² = 88%, *P* < 0.05). Sensitivity analysis suggested the study by Wang et al. (2016) as a potential source of heterogeneity. After excluding this study, the meta-analysis was repeated. The heterogeneity was reduced (I² = 0%, *P* = 0.42), indicating no statistically significant residual heterogeneity. Based on these findings, a fixed-effect model was applied for the sensitivity analysis. The pooled results suggested that elevated uric acid levels may be associated with increased odds of CSF (Z = 4.87, *P* < 0.05). Specifically, each increase in uric acid level was associated with 1.90-fold higher odds of CSF (95% CI: 1.47–2.46).

In addition to the eight factors included in the quantitative meta-analysis, a wide range of additional clinical, biochemical, hematological, and imaging-related variables were identified across the included studies and summarized separately because of insufficient data for pooling. These non-pooled factors were typically reported in only one or two studies, which precluded reliable quantitative synthesis. Despite this limitation, many of these variables—including inflammatory markers, lipid-related indices, hematologic parameters, metabolic indicators, and cardiac functional measurements—showed a possible tendency toward increased odds of coronary slow flow (CSF) in individual studies. Collectively, these findings suggest that CSF may be a multifactorial condition involving inflammatory activation, metabolic dysregulation, hemorheological changes, and microvascular dysfunction. However, given the limited number of studies and lack of standardized definitions for most variables, these associations should be interpreted as exploratory and hypothesis-generating rather than confirmatory evidence (Table 3).

**Table 3:** Summary of risk factors identified in the included studies but not eligible for quantitative meta-analysis

Factor	Study	Effect Estimate	95% CI	Main Finding	Reason Not Pooled
ET-1	Li Bing 2019	OR = 3.765	2.521 ~ 6.571	Increased CSF odd	Only 2 studies
	Wang Jirong 2015	OR = 1.114	1.080 ~ 1.565		
Smoking duration	Wang Shuai 2016	OR = 1.028	1.017 ~ 1.038	Increased CSF odd	Single study
Heart rate	Wang Shuai 2016	OR = 0.987	0.978 ~ 0.997	Decreased CSF odds	Single study
hs-CRP/Alb	Sun Yang 2021	OR = 1.543	1.000 ~ 2.381	Increased CSF odd	Single study
Tp–Te interval	Lei Jing 2022	OR = 4.356	1.834 ~ 10.348	Increased CSF odd	Single study
Tp–Te/QT ratio	Lei Jing 2022	OR = 4.075	1.554 ~ 10.683	Increased CSF odd	Single study
HCT,	Lu Cong 2018	OR = 1.083	1.020 ~ 1.150	Increased CSF odd	Only 2 studies
	Kong Qianwen 2020	OR = 1.54	1.127 ~ 2.110		
LDL-C	Chen Yun 2024	OR = 1.488	1.006 ~ 2.202	Increased CSF odd	Single study
SII	Chen Yun 2024	OR = 1.004	1.002 ~ 1.007	Increased CSF odd	Single study
TyG index	Chen Yun 2024	OR = 1.65	1.048 ~ 2.597	Increased CSF odd	Only 2 studies
	Nan Jing 2025	OR = 2.76	1.43 ~ 4.84		
Diabetes mellitus	Yu Jinag 2024	OR = 2.654	1.112 ~ 6.338	No clear association	measures differed
	Yang Qianqian 2017	OR = 0.253	0.075 ~ 0.858		
Hyperlipidemia	Yu Jinag 2024	OR = 2.349	1.191 ~ 4.631	Increased CSF odd	Only 2 studies
	Kenan Toprak 2024	OR = 1.090	1.072 ~ 1.922		
Coronary sinus diameter	Yu Jinag 2024	OR = 1.097	1.015 ~ 1.185	Increased CSF odd	Single study
Left atrial left–right diameter	Yu Jinag 2024	OR = 1.091	1.013 ~ 1.174	Increased CSF odd	Single study
E wave	Yu Jinag 2024	OR = 1.091	1.013 ~ 1.174	Increased CSF odd	Single study
Red blood cell count	Zhao Ziwei 2020	OR = 2.039	1.378 ~ 3.017	Increased CSF odd	Single study
Lp(a)	Zhu Yanan 2023	OR = 1.107	1.005 ~ 1.265	Increased CSF odd	Single study
Cystatin C,	Zhu Yanan 2023	OR = 1.369	1.014 ~ 2.05	Increased CSF odd	Single study
MCV	Wang Jing 2015	OR = 0.871	0.779 ~ 0.974	Decreased CSF odds	Single study
Mitral inflow E/A ratio < 1	Zhang Zhiqiang 2025	OR = 1,840	1.109 ~ 3.053	Increased CSF odd	Single study
P-selectin	Zhang Zhiqiang 2025	OR = 1,446	1.005 ~ 2.080	Increased CSF odd	Single study
BNP	Zhang Zhiqiang 2025	OR = 1,13	1.041 ~ 1.227	Increased CSF odd	Single study
NLR	Zhang Yuxin 2015	OR = 3,448	1.065 ~ 11.165	Increased CSF odd	Only 2 studies
	Qin Gang 2017	OR = 3,249	1.895 ~ 5.323		
LMR	Yang Qianqian 2017	OR = 0.683	0.537 ~ 0.868	Decreased CSF odds	Single study
NMR	Yang Qianqian 2017	OR = 1.631	1.230 ~ 2.162	Increased CSF odd	Single study
Age	Wang Shuai 2016	OR = 1.044	1.031 ~ 1.056	Increased CSF odd	Single study
Total bile acids	Yang Qianqian 2017	OR = 0.903	0.844 ~ 0.965	Decreased CSF odds	Single study
GDF-15	Yao Kunmei 2023	OR = 1.004	1.002 ~ 1.006	Increased CSF odd	Single study
RDW	Xumin 2016	OR = 2.114	1.095 ~ 4.000	Increased CSF odd	Only 2 studies
	Wang Li 2021	OR = 2.266	1.087 ~ 4.096		
fQRS	Yang Fan 2016	OR = 3.620	1.382 ~ 8.332	Increased CSF odd	Only 2 studies
	Kong Qianwen 2020	OR = 2.331	1.765 ~ 8.213		
Hypertension combined with diabetes mellitus	Chen Bo 2021	OR = 4.290	1.250 ~ 8.620	Increased CSF odd	Only 2 studies
Anxiety	Wang Ting 2019	OR = 2.119	1.103 ~ 4.071	Increased CSF odd	Single study
Depression	Wang Ting 2019	OR = 2.165	1.122 ~ 4.179	Increased CSF odd	Only 2 studies
	Ahmed A. Elamragy 2019	OR = 1.200	1.100 ~ 1.300		
sLOX-1	Yan Jie 2018	OR = 1.260	1.080 ~ 1.430	Increased CSF odd	Only 2 studies
	Ren Yingang 2016	OR = 2.044	1.274 ~ 3.279		
Leptin	Yan Jie 2018	OR = 1.040	0.940 ~ 1.360	No clear association	Single study
LV global longitudinal strain	Ahmed Shawky Shereef 2024	OR = 2.200	1.57 ~ 3.09	Increased CSF odd	Single study
Eosinophil count	Qin Gang 2017	OR = 1.082	1.002 ~ 1.007	Increased CSF odd	Only 2 studies
	Wang Li 2021	OR = 7.767	4.634 ~ 12.350		
PDW	Kong Qianwen 2020	OR = 1.974	1.540 ~ 5.490	Increased CSF odd	Single study
D-dimer,	Ge Hailong 2015	OR = 1.532	1.018 ~ 2.105	Increased CSF odd	Single study
miR-126	Wang Lei 2019	OR = 2.513	1.245 ~ 1.387	Increased CSF odd	Single study
miR-155	Yang Pengkang 2020	OR = 1.597	1.109 ~ 2.301	Increased CSF odd	Single study
Erythrocyte aggregation index	Yang Pengkang 2020	OR = 2.654	1.348 ~ 5.225	Increased CSF odd	Single study
Plasma thrombomodulin	Tuo Buxiong 2018	OR = 1.980	1.020 ~ 3.561	Increased CSF odd	Single study
Serum endocan	Han Wei 2015	OR = 2.312	1.329 ~ 4.019	Increased CSF odd	Single study
PAF	Shi Zhifang 2023	OR = 1.031	1.018 ~ 1.044	Increased CSF odd	Single study
Lp-PLA2	Shi Zhifang 2023	OR = 1.060	1.039 ~ 1.082	Increased CSF odd	Single study
Female sex	Li Shijing 2018	OR = 0.541	0.308 ~ 0.950	Decreased CSF odds	Single study
Fundus arteriosclerosis grade > 2	Li Shijing 2018	OR = 0.453	0.250 ~ 0.821	Decreased CSF odds	Single study
Neutrophil percentage-to-albumin ratio	Tu Guang 2023	OR = 8.067	1.212 ~ 53.691	Increased CSF odd	Single study
NHR	Tu Guang 2023	OR = 2.211	1.582 ~ 3.089	Increased CSF odd	Single study
Triglycerides	Ramy Raymond Elias 2025	OR = 7.729	1.251 ~ 47.738	Increased CSF odd	Only 2 studies
	Ahmed A. Elamragy 2019	OR = 1.010	1.000 ~ 1.020		
Serum glucose	Ramy Raymond Elias 2025	OR = 20.291	2.611 ~ 157.687	Increased CSF odd	Single study
BUN	Ramy Raymond Elias 2025	OR = 12.057	1.827 ~ 79.566	Increased CSF odd	Single study
Serum osmolality	Ramy Raymond Elias 2025	OR = 83.119	4.488 ~ 1539.245	Increased CSF odd	Single study
NHHR	Kenan Toprak 2024	OR = 1.054	1.037 ~ 1.071	Increased CSF odd	Single study
Atherogenic index	Kenan Toprak 2024	OR = 1.049	1.025 ~ 1.074	Increased CSF odd	Single study
Castelli risk index II	Kenan Toprak 2024	OR = 1.157	1.061 ~ 1.458	Increased CSF odd	Single study
Whole blood viscosity	Mehmet Serkan Cetin 2015	OR = 2.794	2.103 ~ 3.711	Increased CSF odd	Single study
WBC	Mehmet Serkan Cetin 2015	OR = 1.206	1.039 ~ 1.400	Increased CSF odd	Single study
GLS	Yunbo Zhang 2025	OR = 1.339	1.112 ~ 1.613	Increased CSF odd	Single study
Global left ventricular perfusion index	Yunbo Zhang 2025	OR = 0.456	0.209 ~ 0.994	Decreased CSF odds	Single study
Hemoglobin.	Hamidreza Sanati 2016	OR = 1.400	1.100 ~ 1.900	Increased CSF odd	Single study

### Publication bias analysis

**Fig. 3:**
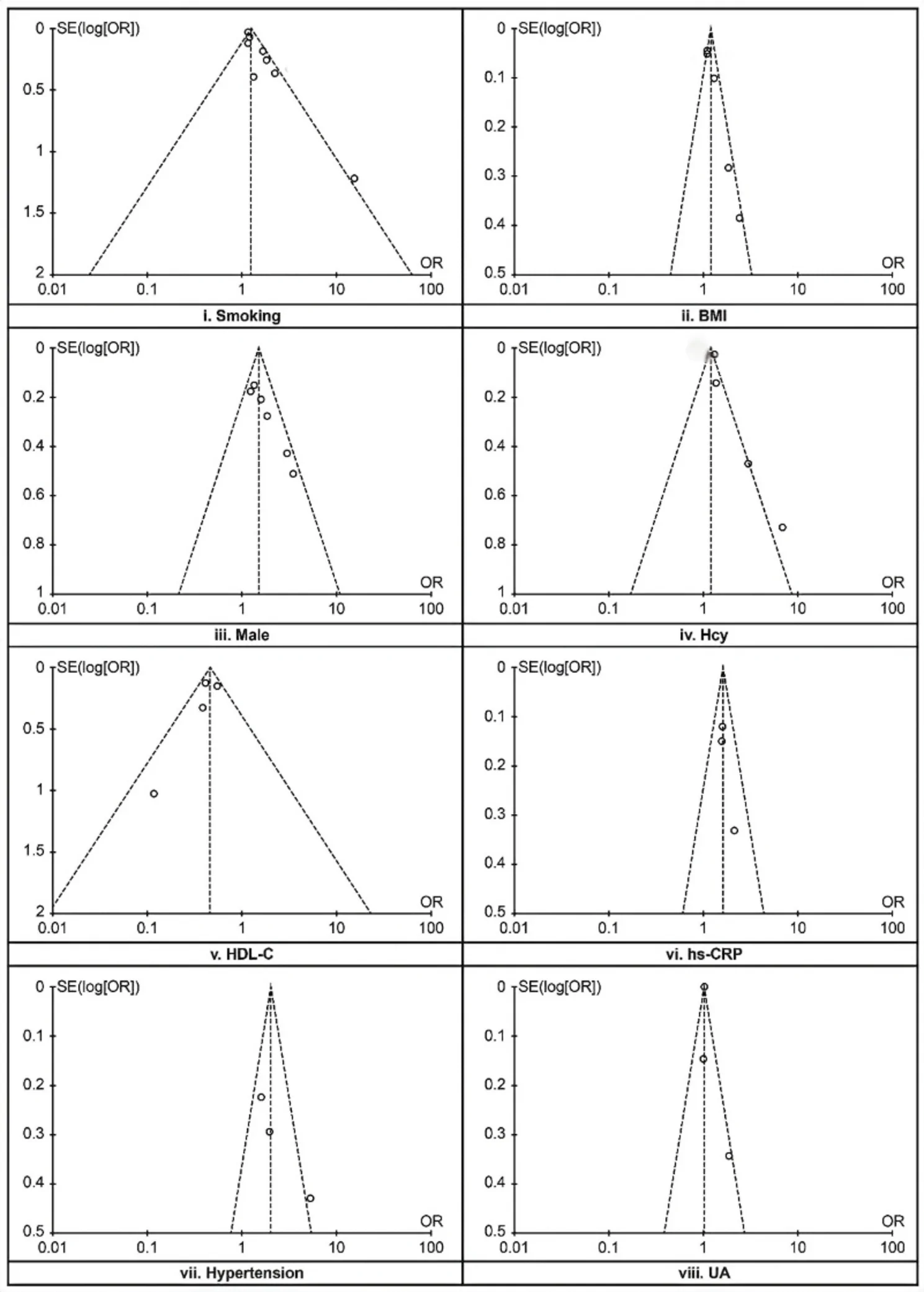
Funnel plots for the meta-analysis of factors associated with coronary slow flow

Publication bias was assessed using funnel plots. Funnel plots were generated for smoking, body mass index (BMI), male sex, homocysteine, high-density lipoprotein cholesterol (HDL-C), high-sensitivity C-reactive protein (hs-CRP), hypertension, and uric acid. The effect estimates in the funnel plots were generally clustered around the pooled effect sizes, with most studies located in the upper region. Visual inspection showed no obvious unilateral gaps or systematic deviations in the distribution of scatter points. However, a small number of studies with limited sample sizes showed slight deviations near the lower part of the funnel, suggesting possible mild asymmetry, which may be related to small-study effects, differences in study design, or selective publication of positive findings. Because most outcomes included fewer than 10 studies, funnel plots were interpreted as exploratory visual assessments rather than definitive evidence of publication bias (see Fig. 3).

## Discussion

This meta-analysis included case–control studies published between 2015 and 2025. The results suggested that smoking, elevated body mass index (BMI), male sex, hypertension, and higher levels of homocysteine, high-sensitivity C-reactive protein (hs-CRP), and uric acid were associated with higher odds of coronary slow flow (CSF). In contrast, higher levels of high-density lipoprotein cholesterol (HDL-C) were associated with lower odds of CSF. Overall, traditional lifestyle-related and cardiometabolic factors may interact with inflammatory- and oxidative stress–related biochemical markers in the development of CSF. Higher HDL-C levels may be associated with a lower likelihood of CSF; however, this finding should be interpreted cautiously because moderate-to-high heterogeneity remained in several pooled analyses.

The sensitivity analyses suggested that the pooled results for certain variables, such as male sex, were sensitive to the exclusion of specific studies. These studies differed in population characteristics, study design, or adjustment strategies, which may partly explain the observed heterogeneity. Therefore, the stability of these associations should be interpreted with caution, and no causal inference can be drawn. This meta-analysis suggested that smoking was associated with higher odds of coronary slow flow (CSF) (OR = 1.21), which is consistent with previous findings. A case–control study conducted by Sun et al. in 2024 involving 112 patients with CSF reported a history of smoking as an independent factor associated with CSF through multivariable logistic regression analysis. Smoking was often accompanied by unhealthy dietary habits and insufficient sleep, which may further contribute to adverse cardiovascular effects [51]. The mechanisms by which smoking may contribute to CSF are thought to involve tobacco-induced oxidative stress, inhibition of endothelial nitric oxide synthase (eNOS) activity, reduced bioavailability of nitric oxide (NO), and coronary microvascular spasm. These pathophysiological changes may increase distal coronary resistance and subsequently slow coronary blood flow [52]. This meta-analysis suggested that male sex may be associated with higher odds of coronary slow flow (CSF) (OR = 1.51). A study conducted by Elias et al. in 2025 also reported male sex as an independent factor associated with CSF, with an odds ratio of approximately 2.58 (95% CI: 1.12–5.95), suggesting a larger effect estimate than that observed in our pooled analysis. Previous studies have reported a predominance of CSF among young and middle-aged men. This association may be partly explained by a higher prevalence of smoking, greater visceral fat accumulation, enhanced androgen–sympathetic nervous system activity, and reduced estrogen-related vascular protection in men [53]. However, some small-sample studies have reported attenuation or loss of statistical significance for sex differences after adjustment for lifestyle-related factors. These findings suggest that male sex may represent a marker of clustered cardiovascular risk factors rather than an isolated biological determinant [54]. The findings of the present meta-analysis may partly explain this heterogeneity across studies. Regarding body mass index (BMI), this meta-analysis suggested that each unit increase in BMI was associated with higher odds of coronary slow flow (CSF) (OR = 1.20). Several single-center studies have similarly reported that overweight and obesity are associated with CSF. In some of these studies, BMI was included in regression models as a categorical variable, and overweight or obese individuals had approximately 1.5–2.0 times higher odds of CSF than individuals with normal weight [55]. In contrast, an earlier case–control study by Sanati et al. reported that lower BMI was associated with CSF and suggested that this finding may reflect a chronic catabolic state or subclinical myocardial injury [56]. However, this result is inconsistent with the majority of available evidence, suggesting considerable heterogeneity across studies in terms of patient selection, baseline characteristics, and BMI categorization. Consequently, the true impact of obesity on CSF may have been underestimated in certain populations. Taken together, obesity-related insulin resistance, enhanced sympathetic nervous system activity, chronic low-grade inflammation, and dyslipidemia may contribute to coronary microvascular remodeling and early atherosclerotic changes. These pathophysiological changes may increase coronary microcirculatory resistance and contribute to the development of slow coronary flow [57]. In the present meta-analysis, hypertension was associated with higher odds of coronary slow flow (CSF) (OR = 2.32). Several studies have reported a higher prevalence of hypertension among patients with CSF. Multivariable analyses have also reported hypertension as an independent factor associated with CSF. Previous invasive coronary function studies have suggested that coronary flow reserve may already be reduced during the prehypertensive stage. Chronic pressure overload may lead to structural remodeling of small arteries, including smooth muscle hypertrophy, increased wall thickness, and luminal narrowing. These changes may result in persistently elevated distal vascular resistance and reduced microvascular compliance, thereby contributing to a slow-flow phenotype, particularly under stress or during physical exertion [58]. In this meta-analysis, elevated homocysteine (Hcy) levels were associated with higher odds of coronary slow flow (CSF) (OR = 1.55). A meta-analysis published in PLOS ONE in 2023 by Yu et al. included 13 studies comprising 625 patients with CSF and 550 controls and reported higher Hcy levels in the CSF group, with a pooled standardized mean difference (SMD) of approximately 1.45 (95% CI: 0.94–1.96). The authors further noted that the association between Hcy and CSF appeared more pronounced when the mean TIMI frame count was ≥ 46 [59]. Earlier evidence from Erbay et al. (2004) also reported higher plasma Hcy levels in patients with CSF than in controls [60]. Compared with these findings, the present meta-analysis treated Hcy as a continuous variable and pooled adjusted odds ratios, resulting in a slightly lower effect estimate but a consistent direction of association. These findings further support a potential association between Hcy and CSF. Mechanistically, elevated Hcy may induce oxidative stress, reduce nitric oxide (NO) bioavailability, and increase reactive oxygen species production, thereby impairing endothelial function and contributing to a hypercoagulable state. These alterations may contribute to coronary microvascular dysfunction and the development of slow coronary flow. In the present meta-analysis, high-sensitivity C-reactive protein (hs-CRP) was associated with higher odds of coronary slow flow (CSF) (OR = 1.62), suggesting a potential association between systemic inflammation and CSF. A 2024 study by Sun et al. reported higher hs-CRP levels in patients with CSF than in controls. hs-CRP remained independently associated with CSF in multivariable analysis. Previous studies by Turhan et al. also reported elevated plasma levels of intercellular adhesion molecule-1 (ICAM-1), vascular cell adhesion molecule-1 (VCAM-1), and E-selectin in patients with CSF, suggesting that endothelial activation may be involved in the pathogenesis of CSF [61]. The findings of this meta-analysis provide population-level evidence suggesting that CSF may represent an inflammation-related microvascular syndrome. In addition, our pooled analysis suggested a positive association between uric acid levels and higher odds of CSF (OR = 1.90), further suggesting that metabolic and oxidative stress–related pathways may be involved in CSF. Several recent studies have further explored the relationship between uric acid–related biomarkers and coronary slow flow (CSF). A study by Demirkıran et al., published in Annals of Medical Research in 2023, reported that the serum uric acid-to-albumin ratio (UAR) was independently associated with CSF. In that study, elevated uric acid alone was associated with an odds ratio (OR) of approximately 2.22 (95% CI: 1.55–3.20). In contrast, the OR for UAR was approximately 37.7, suggesting a stronger association with CSF [62]. Similarly, Zhang et al. (2024), in a study published in BMC Cardiovascular Disorders, reported that UAR increased with the number of affected coronary vessels. A UAR value > 7.9 was associated with CSF, with an area under the curve (AUC) of approximately 0.88, suggesting good discriminatory performance [63]. These findings complement the results of the present meta-analysis. Although elevated uric acid alone was associated with higher odds of CSF, composite indices incorporating uric acid with albumin or high-density lipoprotein cholesterol (HDL-C), which may reflect combined inflammatory and oxidative stress burden, appear to have stronger associations with CSF. From a pathophysiological perspective, elevated uric acid levels are often accompanied by increased generation of reactive oxygen species and reduced antioxidant capacity. These changes may suppress nitric oxide (NO) production, exacerbate endothelial dysfunction, and contribute to microvascular atherosclerosis and microthrombus formation, thereby impairing coronary microcirculation [64]. In contrast to the aforementioned factors, this meta-analysis suggested that elevated high-density lipoprotein cholesterol (HDL-C) levels were associated with lower odds of coronary slow flow (CSF) (OR = 0.45). Several studies have reported that low HDL-C remains independently associated with CSF even after adjustment in multivariable models. A case–control study by Aciksari et al. (2022) reported that an increased triglyceride-to-HDL-C ratio was associated with CSF. This finding suggests that an “atherogenic lipid profile,” characterized by high triglycerides and low HDL-C, may be commonly observed in patients with CSF [65]. HDL-C exerts protective vascular effects through multiple mechanisms, including promotion of reverse cholesterol transport, enhancement of endothelial nitric oxide (NO) production, and anti-inflammatory and antioxidant properties. These mechanisms help maintain endothelial homeostasis and preserve microvascular function [66]. The findings of the present meta-analysis provide population-level evidence supporting the protective role of HDL-C in CSF and offer a theoretical basis for future interventions aimed at improving HDL-C levels or functional capacity. In addition to the factors included in the quantitative meta-analysis, several other clinical, biochemical, hematological, inflammatory, psychological, and imaging-related variables were reported in the included studies. These factors were not pooled because they were generally reported in only one or two studies, or because differences in measurement methods, reporting formats, or variable definitions limited the comparability of effect estimates. Although many of these variables showed potential associations with coronary slow flow (CSF) in individual studies, the current evidence remains insufficient to support firm conclusions.

Most of the factors identified in this study converge on a common pathophysiological axis involving endothelial dysfunction, microcirculatory impairment, and activation of prothrombotic and inflammatory pathways. Long-standing hypertension, obesity, and metabolic syndrome may contribute to arterial remodeling, increasing vascular resistance and reducing coronary flow reserve, which may predispose to a slow-flow phenotype. Smoking, hyperuricemia, elevated homocysteine, and low HDL-C may contribute to oxidative stress, endothelial dysfunction, and impaired vasodilation. Elevated hs-CRP and inflammatory indices, such as the uric acid-to-albumin ratio (UAR) and uric acid-to-HDL-C ratio (UHR), may reflect persistent systemic inflammation and endothelial activation, which is consistent with a prothrombotic phenotype in CSF. However, the existing literature also contains areas of controversy. First, a few studies have suggested that low BMI or “normal weight” may be associated with CSF [67], which contrasts with our findings and the predominant evidence suggesting higher odds of CSF with overweight or obesity. This discrepancy may reflect selection bias, small-study effects, or the limitations of BMI in capturing visceral adiposity and body fat distribution. Second, some studies have reported attenuated or nonsignificant associations for uric acid or hs-CRP after rigorous adjustment for components of metabolic syndrome, suggesting that part of these associations may be mediated through metabolic syndrome rather than representing fully independent factors associated with CSF [68]. Moreover, the 2023 meta-analysis by Yu et al. also reported substantial heterogeneity in the effect estimates for homocysteine, with some cohorts failing to detect significant differences. Collectively, these inconsistencies suggest that CSF is unlikely to be attributable to a single etiological pathway. Rather, it may represent a shared clinical phenotype arising from complex interactions among multiple associated factors in susceptible individuals. This meta-analysis suggests that many factors associated with coronary slow flow (CSF) are potentially modifiable, which may provide opportunities for clinical intervention. Early intervention—particularly smoking cessation, weight control, dietary optimization, and regular physical activity—may help reduce the likelihood of CSF. However, further research is needed to clarify the relative importance of different factors, especially considering the potential influence of non-modifiable factors, such as sex and genetics. Optimizing blood pressure and metabolic control: In patients with concomitant hypertension, dyslipidemia, hyperuricemia, or hyperhomocysteinemia, management in accordance with current clinical guidelines may be considered. When appropriate, folic acid and B-vitamin supplementation, urate-lowering therapy, and intensified lipid-lowering strategies, such as statins or ezetimibe, may be considered as part of individualized management. Assessment of inflammation and endothelial function: In centers with adequate resources, composite inflammatory and endothelial-related indices—such as hs-CRP, neutrophil-to-lymphocyte ratio (NLR), monocyte-to-HDL-C ratio (MHR), systemic immune-inflammation index (SII), and uric acid–based ratios, such as UAR and UHR—may facilitate more refined clinical assessment and monitoring in patients with CSF. Multidisciplinary management: CSF may coexist with anxiety, depression, and other forms of psychological stress. Integrating psychosomatic interventions may help alleviate symptom burden and improve quality of life. However, high-quality randomized controlled trials evaluating such strategies remain limited and are needed in future research.

This study has several limitations that should be acknowledged. First, most of the included studies were single-center case-control investigations conducted predominantly in Asian populations, with a notable concentration of studies from China. This geographic bias may influence the magnitude of risk estimates and limit the generalizability of these findings to other regions. While ethnic and regional differences in the distribution of risk factors for coronary slow flow (CSF) are acknowledged, the results may not be fully representative of populations outside Asia. However, given the scarcity of data on CSF from non-Asian populations, the current analysis provides valuable insights based on the available literature. Future studies involving diverse ethnic and geographic groups are needed to confirm the observed associations and assess their broader applicability. Second, this meta-analysis showed substantial heterogeneity across several key factors, such as smoking (I² = 76%), body mass index (BMI) (I² = 95%), male sex (I² = 86%), and others. The high heterogeneity observed in these analyses suggests considerable variation in study outcomes, which may be influenced by differences in study populations, diagnostic criteria, and methodological approaches. For instance, in studies assessing smoking, variations in the definitions of smoking intensity, participant characteristics, such as age, sex, and comorbidities, and residual confounding could contribute to the observed variability. Similarly, BMI-related studies showed high heterogeneity, likely due to differences in BMI categorization, sample size, and regional population characteristics. Although sensitivity analyses were performed to explore influential studies and assess heterogeneity (e.g., after excluding specific studies, heterogeneity in smoking and BMI analyses decreased from 76% to 46% and from 95% to 57%, respectively), moderate-to-high heterogeneity remained in several pooled estimates. This highlights the need for caution when interpreting pooled results across heterogeneous study designs and populations. To account for between-study heterogeneity, a random-effects model was applied when appropriate. However, the persistence of moderate-to-high heterogeneity for some factors suggests that additional methods, such as meta-regression, may help explore sources of variation and refine the assessment of associations between these factors and CSF. Further studies with more homogeneous populations and standardized diagnostic criteria are needed to improve the consistency of findings and better inform clinical practice. Third, variations existed across studies in CSF diagnostic criteria—particularly the threshold for TIMI frame count—as well as control selection strategies and the extent of adjustment for confounding factors. These methodological differences made it difficult to fully explain statistical heterogeneity. Fourth, this meta-analysis primarily included traditional and routinely available clinical factors. Emerging biomarkers that have attracted increasing attention in recent years—such as long non-coding RNAs (lncRNAs), circulating cell-free DNA (cfDNA), salusin-α/β, lipoprotein-associated phospholipase A2 (Lp-PLA2), monocyte-to-HDL-C ratio (MHR), and systemic immune-inflammation index (SII)—were not quantitatively synthesized in this analysis. Their independent associations with the development and prognosis of CSF require further high-quality investigation. Future research may focus on several directions. First, large-scale, multicenter prospective cohort studies are needed. These studies should incorporate invasive coronary functional assessments, such as coronary flow reserve (CFR) and index of microcirculatory resistance (IMR), as well as advanced imaging techniques to examine potential causal relationships between identified factors and CSF from both structural and functional perspectives. Second, multidimensional predictive models integrating clinical characteristics, metabolic and inflammatory markers, genetic and epigenetic information, and novel biomarkers should be developed to improve clinical assessment and individualized management strategies for CSF. Third, randomized controlled trials evaluating comprehensive interventions—such as smoking cessation, blood pressure control, urate-lowering therapy, correction of hyperhomocysteinemia, and strategies targeting inflammation and endothelial dysfunction—are needed to assess their effects on symptom burden, hospitalization rates, and major adverse cardiovascular events (MACEs) in patients with CSF. Such studies may provide more robust evidence to support the standardized management of CSF.

Overall, this meta-analysis quantitatively summarizes the associations between CSF and traditional cardiovascular factors, as well as inflammatory and oxidative stress–related biomarkers. The findings are broadly consistent with recent research and suggest that CSF may represent a multifactorial disorder involving coronary microvascular and endothelial dysfunction. This understanding may provide a theoretical basis for comprehensive prevention and management strategies, including lifestyle modification, blood pressure and metabolic control, and interventions aimed at reducing inflammation and improving endothelial function in clinical practice.

## Supplementary Information

Supplementary Material 1.

Supplementary Material 2.

Supplementary Material 3.

Supplementary Material 4.

## Data Availability

All data used in this meta-analysis are publicly available. The datasets used in this study are available in online repositories, and the names of these repositories can be found in the article.
