# Supplementary material for: Clinical risk factors associated with coronary slow flow: a meta-analysis
Source: BMC Cardiovasc Disord. 2026 Jul 29;26:981. doi: 10.1186/s12872-026-06373-9 (PMC13712074; doi:10.1186/s12872-026-06373-9)
Supplement: Supplementary file 4 [file 12872_2026_6373_MOESM4_ESM.docx]

Search strategy

(((((slow coronary flow phenomenon[Title/Abstract]) OR slow coronary flow[Title/Abstract]) OR SCF[Title/Abstract]) OR CSF[Title/Abstract])) AND ((((("Risk factors"[Mesh] OR clinical risk factors[Title/Abstract]) OR risk factor[Title/Abstract]) OR predictors[Title/Abstract]) OR related factors[Title/Abstract]) OR influence factors[Title/Abstract]
