# Supplementary material for: Clinical risk factors associated with coronary slow flow: a meta-analysis
Source: BMC Cardiovasc Disord. 2026 Jul 29;26:981. doi: 10.1186/s12872-026-06373-9 (PMC13712074; doi:10.1186/s12872-026-06373-9)
Supplement: Supplementary file 5 [file 12872_2026_6373_MOESM3_ESM.docx]

**PRISMA 2020 Flow Diagram**

## Identification

## Eligibility

## Included

## Screening
